# Gene Polymorphism and Total Genetic Score in Martial Arts Athletes with Different Athletic Qualifications

**DOI:** 10.3390/genes13091677

**Published:** 2022-09-19

**Authors:** Anna Vostrikova, Victoria Pechenkina, Maria Danilova, Svetlana Boronnikova, Ruslan Kalendar

**Affiliations:** 1 Faculty of Biology, Perm State University, Bukireva, 15, 614990 Perm, Russia; 2 National Laboratory Astana, Nazarbayev University, Nur-Sultan 010000, Kazakhstan; 3 Institute of Biotechnology HiLIFE, University of Helsinki, 00029 Helsinki, Finland

**Keywords:** the renin-angiotensin and the kallikrein-kinin systems, endurance, athletes, sports qualification, total genetic score

## Abstract

The personalized approach in sports genetics implies considering the allelic variants of genes in polymorphic loci when adjusting the training process of athletes. The personalized approach is used both in sports genetics and in medicine to identify the influence of genotype on the manifestations of human physical qualities that allow to achieve high sports results or to assess the impact of genotype on the development and course of diseases. The impact of genes of the renin-angiotensin and kinin-bradykinin systems in the development of cardiovascular disease in athletes has not been defined. This study aims to determine the polymorphisms of four genes (*ACE*, *BDKRB2*, *PPARGC1A* and *NOS3*) and the total genetic score to reveal the predisposition to the formation of physical qualities in martial arts athletes with different athletic abilities. The products of these four genes are involved in the control of blood pressure. The allelic variants of these genes are associated with the development of the physical quality “endurance” and have an indirect influence on the formation of speed and power qualities. The total genetic score (TGS: from 0 to 100 arbitrary units) was calculated from the genotype score in each polymorphism. The athletes were divided into Group I with high and Group II with low qualifications depending on their sports success. Single nucleotide polymorphisms (SNPs) are identified through restriction endonucleases cleavage for PCR amplicons for discriminating between alleles of the target genes *ACE* (rs4646994), *BDKRB2* (rs5810761), *PPARGC1A* (rs8192673) and *NOS3* (rs1799983). Significant differences between the allelic variants of target genes and athletic ability were found between Group I and Group II for genotype G/G of *NOS3* gene and genotypes Gly/Gly and Gly/Ser of *PPARGC1A* gene. The data obtained confirm that athletes with unfavourable genotypes are excluded in the screening phase because their endurance is not fully developed to the required level in martial arts. Martial arts athletes with the highest TGS have the highest skill level. Polymorphic loci of four genes whose products are involved in blood pressure control (*ACE, BDKRB2*, *NOS3* and *PPARGC1A*) can be used in martial arts not only to determine predisposition to cardiovascular disease but also to predispose to the development of speed and power qualities and endurance. The total genetic score can serve as a tool for predicting athletic success.

## 1. Introduction

A personalized approach in sports genetics involves considering allelic variants of genes in polymorphic loci when adjusting the training process of athletes. Similarly, in personalized medicine, the patient is treated according to his genotype. Unfortunately, a personalized approach is rarely used in both sports genetics and medicine. One of the reasons is the lack of a mechanism for identifying the influence of the genotype on the manifestations of the physical qualities of an individual, which allow for achieving high sports results or assessing the influence of the genotype on the development and course of diseases.

More than 200 gene polymorphisms are associated with one form or another of a person’s physical performance and thus with his health [1]. Some studies are devoted to predicting the risk of developing diseases using combinations of appropriate gene polymorphisms [2]. Other studies are aimed at assessing genetic predisposition according to individual polygenic profiles. Usually, when working with athletes, gene polymorphisms associated with such physical qualities as “endurance” and “speed/strength” are studied. The combined influence of several polymorphic variants of genes can serve as a marker for identifying athletes who are genetically predisposed to the manifestation of physical qualities “endurance” and “speed/strength” [3,4].

The polymorphism of the genes of the renin-angiotensin and kinin-bradykinin systems of a person plays an important role in intense physical exertion in athletes [5]. The renin-angiotensin system (*RAS*) is a human hormonal system that includes angiotensinogen (*AGT* gene product), angiotensin I (angiotensin II receptor 1 gene—*AGTR1*), angiotensin II (angiotensin II receptor 2 gene—*AGTR2*), renin (a product of the *REN* gene), angiotensin-converting enzyme (*ACE*), and angiotensin-converting enzyme 2 (ACE2). Protein products of the genes of the renin-angiotensin system are important regulators of blood pressure and homeostatic kidney function [6]. Renin acts on angiotensinogen and converts it to angiotensin I. This peptide, in turn, serves as a substrate for the angiotensin-converting enzyme (*ACE* gene product), which converts angiotensin I to angiotensin II. The angiotensin-converting enzyme also inactivates bradykinin, a product of the BDKRB2 gene. The kallikrein-kinin system (*KKS*) system includes kinin, bradykinin, and bradykinin receptor 2, the gene of which is *BKR2* or *BDKRB2*.

Angiotensin-converting enzyme encodes two isozymes: somatic *ACE*, expressed in tissues, and testicular, in the testicles. *ACE* converts angiotensin I to angiotensin II by inactivating bradykinin. Among more than twenty polymorphic variants of the *ACE* gene, the I/D polymorphism (rs4646994) associated with the presence or absence of the Alu repeat is considered the most significant. At the same time, the insertion of the *Alu* repeat leads to a reduced expression of the *ACE* gene. Many studies have been devoted to the study of this polymorphism in patients with arterial hypertension [3,4,7,8,9,10,11].

The bradykinin receptor 2 (*BKR2*) gene encodes a protein involved in vasorelaxation of blood vessels by stimulating the production of endothelial *NO* synthase and its subsequent generation of *NO* [12,13,14]. According to recent studies, T-allele carriers have higher gene expression than C-allele carriers (rs5810761). High gene expression causes the appearance of a large number of receptors per cell. There are observations that alleles C and I are associated with arterial hypertension, as well as with increased endurance in athletes [12,15].

In sports genetics, attention is paid to polymorphisms of the *ACE* and *BDKRB2* genes. The *ACE* gene (*Angiotensin-1 Converting Enzyme*) is mapped at the locus 17q23. There is an I/D-polymorphism of the gene, while in individuals with the D/D-genotype the maximum level of angiotensin-1 converting enzyme in the blood is determined, with the I/I-genotype—the level of *ACE* in the blood is half as high, and in heterozygotes, the level of the blood enzyme is intermediate [2,14]. Also, the D/D genotype of the *ACE* gene is more characteristic of athletes involved in speed-strength sports, and carriers of the I/I genotype are predisposed to performing long-term physical work, that is, to develop the physical quality of “endurance” [16].

The *BDKRB2* gene (*Bradykinin Receptor b2*) is mapped at the 14q32.2 locus. Bradykinin reduces vascular tone, promotes repair processes, and has an insulin-like effect. Studies have shown that athletes carrying the −9 allele of the BDKRB2 gene are characterized by better aerobic endurance compared to representatives of the +9/+9 polymorphic variant of this gene. It was also determined that +9/−9 genotype, which is a “balanced” variant of heart rhythm regulation, is associated with the most optimal hemodynamic state in the examined athletes [17].

The functioning of the cardiovascular system is influenced by the products of the *NOS3* and *PPARGC1A* genes. The *NOS3* (*Nitric Oxide Synthase 3*) gene is mapped on the long arm of the 7th chromosome (7q36), and consists of 26 exons, encodes an enzyme-endothelial *NO* synthase, which catalyses the formation of nitric oxide (*NO*) from L-arginine. The *NOS3* gene plays an important role in the regulation of the tone of blood vessels, in the functioning of the smooth muscle of the vascular wall, and in the processes of blood coagulation. Polymorphisms of the *NOS3* gene may be involved in the progression of diseases in the cardiovascular system and may be the reasons that contribute to the deterioration of the functions of impulse conduction along the conduction system of the heart muscle. There are 3 genotypes of the *NOS3* G894T polymorphism: G/G, G/T, and T/T (rs1799983). Numerous studies have shown the significance of the corresponding genotypes in descending order for the quality of “endurance”: G/G, G/T, and T/T. Analysis of the composition of muscle fibres showed that carriers of the G allele (G/G and G/T genotypes) have an increased proportion of slow muscle fibres [18].

The *PPARGC1A* (*Peroxisome Proliferator-Activated Receptor Gamma Coactivator 1-Alpha*) gene is localized at the 4p15.1 locus and is expressed predominantly in skeletal muscles of slow muscle fibres, myocardium, brown fat, and kidneys [19]. The most significant polymorphic position of the *PPARGC1A* gene is a single nucleotide polymorphism that results in the substitution of the amino acid glycine for serine at position 482, Gly482Ser (rs8192673). Polymorphism is associated with the manifestation of speed-strength qualities, high performance, and muscular and aerobic endurance (Gly). In addition, several studies [20] showed the relationship of the A-allele (Ser) polymorphism with the risk of developing hypertension and an increase in both systolic and diastolic pressure at a young age (up to 50 years).

The products of these four genes are involved in the control of blood pressure. The allelic variants of these genes are associated with the development of the physical quality “endurance” and have an indirect influence on the formation of speed and strength qualities.

The study of the relationship between gene polymorphism and the manifestation of increased physical activity and success of athletes is possible using two groups of martial arts practitioners with different sports qualifications. In sports genetics, a special place is occupied by studies devoted to the study of associations of genes with diseases, including cardiovascular diseases. This is confirmed by the increase in the number of candidate genes presented in the physical activity genetic map [1]. To quantify the combined effect of gene polymorphisms, the “total genetic score” (hereinafter TGS) is used [14].

Thus, the genes of the renin-angiotensin and kinin-bradykinin systems have been studied in sufficient detail, but the mechanism of their polymorphism influence on the success of the sport in martial arts athletes has not been investigated sufficiently, including in the context of cardiovascular diseases in athletes. Besides the speed and power qualities, the formation of endurance plays an important role for masters of martial arts. Knowledge of individual genetic features will help the athlete and his/her coach to formulate a personalized program aimed at significant improvement of physical fitness indicators. In addition, such studies can be used to determine the predisposition to various diseases.

The study aimed to determine the polymorphisms of *ACE*, *BDKRB2*, *PPARGC1A* and *NOS3* genes and the total genetic score (TGS) to identify the predisposition to the formation of physical qualities in martial arts athletes with different athletic skills.

## 2. Materials and Methods

Molecular genetic analysis was performed on 100 acyclic martial arts athletes at the “Perm city Karate Sports School”; “Perm Region Judo and Sambo Sports School”; “Perm city Judo and Sambo Sports School” of the city of Perm in Russia (Supplementary Figures S1–S4; Tables S1 and S2). This work was discussed by the faculty review board and was approved by the ethical committee of the Perm State National Research University. Institutional written informed consent about nationality declaration, DNA extraction and further investigation was signed and obtained from the participating individuals. The sampling for the study included 100 athletes, which was small, but sufficient for the study of distinct polymorphic loci [11]. The age of the subjects ranged from 10 to 16 years old. Acyclic sports include martial arts (karate, sambo, judo), in which such physical qualities as “speed/strength” and “endurance” are important. Combat sports are characterized by different power strain and energy expenditures that depend on the amount of weight lifted, as well as on the dynamics of the fight, and are accompanied by a variety of biochemical changes in the body of athletes [16]. Four genes were selected for this study, including the renin-angiotensin system gene *ACE* and the kinin-bradykinin system gene *BDKRB2*. In addition, the *NOS3* and *PPARGC1A* genes were studied; the products of different polymorphic positions of these genes affect cardiovascular function.

After defining the sports qualification (sport category), two groups of martial arts athletes were formed depending on their sports successes: Group I with high qualifications included 50 athletes in the second and the third adult categories, as well as the CMS and the first junior category; 39 of them were men and 11 women. Group II with low qualifications consisted of 50 sportsmen with the second and the third junior categories, and also the fighters having no sports category; among them 43 men and 7 women. Martial arts are a predominantly male sport, so the samples are dominated by men, and the number of women in both groups is one-fifth. Each group included athletes between the ages of 10 and 16.

We used PCR to detect polymorphisms of genes: I/D polymorphism of I/D polymorphism of the *ACE* gene (*Angiotensin-1 Converting Enzyme*) and +9/−9 polymorphism of the *BDKRB2* gene (*Bradykinin Receptor b2*). Also, an analysis was made of the Gly/Ser polymorphism of the *PPARGC1A* gene (*Peroxisome Proliferator-Activated Receptor Gamma Coactivator 1-Alpha*) and the G/T polymorphism of the *NOS3* gene (*Nitric Oxide Synthase3*) associated with endurance, but not related to the renin-angiotensin and kinin-bradykinin genes systems. Nevertheless, these genes have an impact on the functioning of the cardiovascular system.

The sampling of biological material (buccal epithelium) for genetic analysis was carried out by scraping the epithelial cells of the oral cavity with disposable cytological brushes in the morning, before training. Voluntary consent was obtained from each athlete for the collection of material and the use of some data in scientific generalizations in accordance with the Helsinki Declaration of 1975 and 1983 [21].

DNA was isolated by using the “Sample GS” kit manufactured by DNA-Technology LLC (Protvino, Russia). The concentration of DNA samples was determined using NanoDrop™ 2000 Spectrophotometers (Thermo Fisher Scientific, Waltham, MA, USA). The concentration of DNA samples was levelled to 5 ng/µL. The primer sequences for amplification of polymorphic loci of the four genes selected for study (Table 1) were synthesized at Syntol LLC (Moscow, Russia).

PCR reactions were performed in a 25 µL reaction mixture. Each reaction mixture contained 25 ng of template DNA, 1× PCR buffer with 2.5 mM of MgCl_2_, 0.2 µM of each primer, 0.25 mM of each dNTP, and 1 U of Taq DNA polymerase (Sileks, Russia). PCR amplification was carried out in a MyCycler Thermal Cycler (Bio-Rad, Hercules, CA, USA) under the following conditions: the initial denaturation step at 94 °C for 2 min, followed by 35 amplifications at 94 °C for 20 s, at 50 °C (*PPARGC1A*), 56 °C (*NOS3*) or 62 °C (*BDKRB2*, *ACE)* for 30 s, and 72 °C for 30 s, followed by a final extension of 72 °C for 3 min [22]. The annealing temperature varied between 50 °C and 62 °C depending on the Tm of the primer composition (Table 1) [23]. To detect the polymorphism for *NOS3*, *BDKRB2*, and *ACE* genes, PCR samples were additionally incubated with Eco24I restriction endonuclease (Thermo Fisher Scientific) with restriction site: 5′-GRGCY↑C-3’/3’-C↑YCGRG-5′ [18]. To detect single nucleotide substitutions for *PPARGC1* gene, amplicons were incubated together with MspI restriction endonuclease (Thermo Fisher Scientific) with the restriction site: 5′-C↓CGG-3′/3′-GGC↑C-5′. The reaction mixture was incubated at 37 °C [24].

Allelic variants of PCR amplification products for the genes *BDKRB2*, *ACE*, and *NOS3*, *PPARGC1A* were analysed by electrophoresis in 2% agarose gel stained with ethidium bromide and visualized in the GelDoc XR Bio-Rad (USA) gel documentation system under UV light. The determination of the lengths of DNA fragments was carried out using the Quantity One 4.6.2 (Bio-Rad, USA) program using a molecular weight ladder (50 bp DNA Ladder, SibEnzyme LLC (Moscow, Russia).

For the *ACE* gene, the previously established [25] correspondence of fragments to allelic variants of genes or genotypes was confirmed: the presence of one DNA fragment 190 bp long. corresponded to the D/D genotype; one DNA fragment 477 bp long.–genotype I/I; two fragments (477 and 190 bp)–heterozygote I/D (Supplemental Material). As a result, for the *BDKRB2* gene [22], we confirmed the presence of one 100 bp DNA fragment corresponding to the genotype +9/+9; one DNA fragment 91 bp long–to genotype −9/−9; two fragments (100 and 91 bp)–to heterozygote +9/−9 (Supplemental Material). For the *NOS3* gene, as previously revealed [18], the presence of three fragments (90, 158, and 248 bp) corresponded to the G/T heterozygote, and two fragments (158 and 90 bp) corresponded to the T/T homozygote, one DNA fragment 248 bp long–to genotype G/G (Supplemental Material). For the *PPARGC1A* gene [24], the expression of three DNA fragments (169, 209, and 378 bp) corresponded to the Gly/Ser heterozygote, two DNA fragments 169 and 209 bp long determined the Gly/Gly genotype, one 378 bp DNA fragment corresponding to Ser/Ser homozygous (Supplemental Material).

To assess the genetic predisposition of the tested combatants, the total genetic score method was used [14] based on the polygenic profiles of the physical quality “endurance” that we obtained. Individual profiles of the following polymorphisms of four genes with the assignment of points to their variants (0, 1, 2) were used for the complex evaluation of the “endurance” quality:

1. *ACE* I/D-polymorphism: I/I = 2, I/D = 1, D/D = 0.
2. *BDKRB2* +9/−9- polymorphism: −9/−9 = 2, +9/−9 = 1, +9/+9 = 0.
3. *NOS3*G894T- polymorphism: G/G = 2, G/T = 1, T/T = 0.
4. *PPARGC1A* Gly482Ser polymorphism: Gly/Gly = 2, Gly/Ser = 1, Ser/Ser = 0.

TGS of a polygenic profile associated with the physical quality “endurance”, following the method of A.J. Williams and D.P. Folland [14] was calculated separately for two genes, the first block of which included two genes, namely the gene of the renin-angiotensin system *ACE* and the gene of the kinin-bradykinin system *BDKRB2*. The second block (two genes) for calculating the TGS included the *NOS3* and *PPARGC1A* genes associated with the work of the cardiovascular system, according to the formulas:

TGS “endurance” = (100:4) × (GS *ACE* + GS *BDKRB2*);

TGS “endurance” = (100:4) × (GS *NOS3*+ GS *PPARGC1A*).

Martial arts practitioners with TGS equal to 100 have the highest predisposition to the development of the physical quality “endurance”. Athletes with TGS equal to 0 do not have a genetic predisposition to develop endurance. As noted by coaches from the studied sports schools, athletes with such TGS do not have the level of endurance necessary for practising sambo, karate, or judo and voluntarily end their sports careers.

The analysis of the obtained data was performed using the STATISTICA 6.0 program to determine the normality of the distribution. A comparison of unrelated samples (genotypes of athletes between Group I with high qualification and Group II with low qualification) was carried out by Fisher’s test, Fisher’s F-test standard is 1.96 (*p* = 0.05). Comparison of unrelated samples (TGS of athletes between Group I with high qualification and Group II with low qualification) was also carried out by Fisher’s test, Fisher’s standard F-test is 1.96 (*p* = 0.05). Comparison of TGS frequencies between high-skill Group I and low-skill Group II was performed using a chi-square (χ2) test. Differences between the compared samples were considered statistically significant at *p* = 0.05.

## 3. Results

Molecular genetic analysis of the frequency of the more favourable for the development of physical quality “endurance” genotype I/I of the *ACE* gene (Table 2) showed insignificant differences (F_exp_ 1.20 < 1.96, *p* = 0.05) between Group I with high qualifications (frequency 0, 16) and Group II with low qualifications (frequency 0.08). However, the D/D genotype in Group II occurs significantly more often than in Group I (F_exp_ 2.14 > 1.96, *p* = 0.05).

The differences between Group I and Group II on the gene *BDKRB2* were not significant. By genotype +9/+9 (F_exp_ 1.36 < 1.96 at *p* = 0.05) and also by genotype +9/−9 (F_exp_ 1.36 < 1.96 at *p* = 0.05). At the same time, the −9/−9 genotype, which is the most favourable in terms of physical quality “endurance”, was not determined in this sample.

Differences in the G/G genotype of the NOS3 gene associated with the physical quality “endurance” between Group I with high qualification and Group II with low qualification were significant (F_exp_ 3.39 > 1.96, *p* = 0.05). Also, the less favourable T/T genotype was found not significantly, but more in Group II with low qualification (F_exp_ 1.63 < 1.96, *p* = 0.05).

Analysis of the genotypes of the *PPARGC1A* gene showed significant differences in the favourable Gly/Gly genotype (F_exp_ 2.32 > 1.96, *p* = 0.05). In Group I with high qualifications with this genotype, 40 athletes were identified (frequency 0.80), while in Group II—29 people (frequency 0.58). In addition, there were significant differences in the Gly/Ser genotype (F_exp_ 2.50 > 1.96, *p* = 0.05). The number of athletes with this genotype prevails in Group II-18 people (frequency 0.36) compared to Group I–7 combatants (frequency 0.14). At the same time, there was the same number of athletes with an unfavourable Ser/Ser genotype in both groups: three athletes each (frequency 0.06). The allelic variants of four genes (genotypes) identified in 100 athletes are presented in additional materials.

As a result of the study, it was found that in 100 wrestlers the frequency of occurrence of TGS, calculated for the physical quality “endurance”, based on the polymorphism of four genes, varied from 12 to 75 (Table 3).

Significant differences in TGS between Group I with high qualifications and Group II with low qualifications were observed at TGS equal to 12 (F_exp_ 2.32 > 1.96, *p* = 0.05), TGS equal to 25 (F_exp_ 3.22 > 1.96, p = 0.05) and TGS equal to 62 (F_exp_ 2.27 > 1.96, *p* = 0.05). At the same time, there were no athletes with TGS equal to 75 in group II with low qualifications. A TGS equal to 37 was observed most frequently in the total sampling of martial arts athletes with a frequency of 0.36.

**Figure 1:**
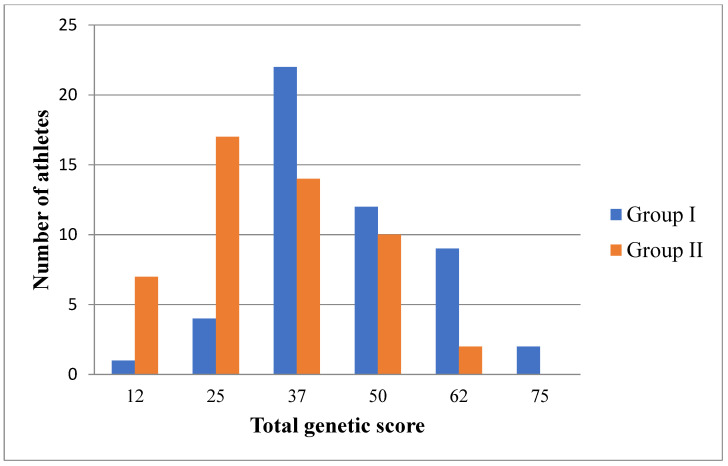
Distribution of the total genetic score established in combatants (*n* = 100) for the quality of “endurance”, based on the polymorphism of the *ACE*, *BDKRB2*, *NOS3*, and *PPARGC1A* genes.

Figure 1 shows the distribution of the TGS index calculated for endurance quality based on *ACE*, *BDKRB2*, *NOS3*, and *PPARGC1A* gene polymorphisms.

A noticeable predominance of high-skilled athletes is observed with TGS equal to 37 and above. Using a chi-square test, statistical significance was determined between the two samples (Group I with high skill and Group II with low skill) and the TGS value. The value of the criterion χ2_emp_ is 20.962, and the critical value of χ2_cr_ at the significance level *p* = 0.01 is 15.086. Thus, the relationship between the two samples and the TGS value is statistically significant at a significance level of *p* < 0.01. This indicates that the calculation of TGS is suitable for assessing the relationship between two variables, namely, allelic variants of the *ACE*, *BDKRB2*, *NOS3*, and *PPARGC1A* genes and TGS found in combatants of different qualifications.

As a result of the study, it was found that in 100 athletes the frequency of occurrence of TGS, calculated for the physical quality “endurance”, based on the polymorphism of the *ACE* and *BDKRB2* genes, varied from 0 to 75 (Table 4). In the general sample, when studying two genes, the most common TGS was 25 in 51 people. With a sample of 100 athletes, the frequency is 0.51. Last of all, in the study sample, the largest TGS was set, equal to 75, in 5 people, naturally with a frequency of 0.05. TGS equal to 0 was determined in 22 people (frequency 0.22), and TGS equal to 50 was found in 22 athletes (frequency 0.22). The highest TGS score of 100 was not found in the total study sample.

In Group I of highly qualified martial arts practitioners, the TGS defined for the quality of “endurance” (Table 4) varied from 0 (for six people, with a frequency of 0.12) to 75 for four athletes (frequency of 0.08). The average value of TGS, equal to 50, was determined in 14 people with a frequency of 0.28. A low TGS value of 25 was found in 26 people in Group I with a frequency of 0.52.

**Figure 2:**
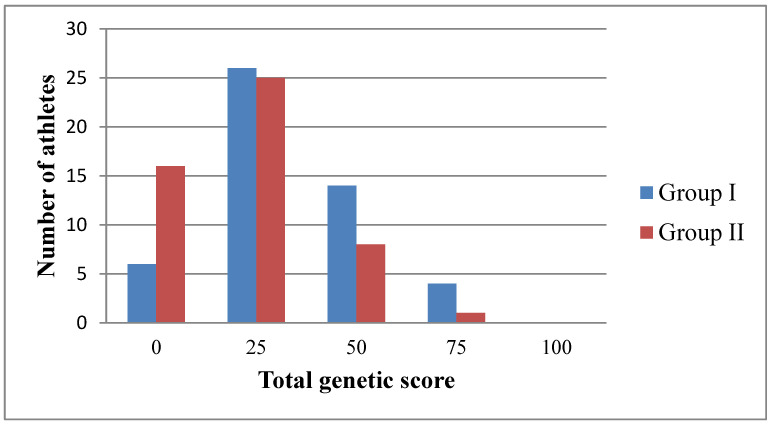
Distribution of the total genetic score established in combatants (*n* = 100) for the quality of “endurance”, based on the polymorphism of the *ACE* and *BDKRB2* genes.

In a sample of 100 athletes, the distribution of the TGS calculated for the quality of “endurance” based on the polymorphism of the *ACE* and *BDKRB2* genes is shown in Figure 2. In Group I with high qualifications, 4 athletes had a high result (TGS = 75), and 14 people had an average score (TGS = 50). The low indicator (TGS = 25) was determined in 26 people, and the lowest (TGS = 0) was found in 6 people. The highest indicator (TGS = 100) is not observed. A significant difference between the two groups of different qualifications on the graph is noticeable in the region of TGS = 0. This indicates that in Group I with high qualifications, there are fewer athletes with the lowest TGS. Starting from the TGS equal to 50, there is a persistent tendency for the number of athletes with a high TGS with high qualifications to prevail over the number of athletes with low qualifications, which indicates that martial arts practitioners with a genetic predisposition to the formation of endurance have reached high qualifications.

In Group II with low qualifications, 1 athlete with a frequency of 0.02 had a high result (TGS = 75). The average indicator (TGS = 50) was found in 8 athletes (frequency 0.16). A low indicator (TGS = 25) was determined in 25 people with a frequency of 0.50. The lowest indicator of TGS = 0 was found in 16 athletes, which is noticeably and significantly higher than in Group I (F_exp_ 2.37 > 1.96 at *p* = 0.05).

Using the Fisher test, an estimate of the differences between the two samples was determined-the total genetic score between groups with high and low qualifications (Table 4). A significant difference was found at TGS = 0 (the lowest indicator), the ratio of 6 people (frequency 0.12) in Group I and 16 people (frequency 0.32) in Group II (F_exp_ 2.37 > 1.96 at *p* = 0.05). In all other cases, no significant differences were found between the values of TGS in the first and second groups.

Using a chi-square test, statistical significance was determined between the two samples (Group I with high skill and Group II with low skill) and the TGS value. The value of the criterion χ2_emp_ is 8.001, and the critical value of χ2_cr_ at the significance level *p* = 0.05 is 7.815. Significance level *p* = 0.046. Thus, the relationship between the two samples and the TGS value is statistically significant at a significance level of *p* < 0.05. This indicates that the calculation of TGS is acceptable for assessing the relationship between two variables, namely, allelic variants of the *ACE* and *BDKRB2* genes and TGS found in combatants of different qualifications.

The second block of genes included data on the TGS calculated for the development of the quality of “endurance” in athletes based on the *NOS3* and *PPARGC1A* polymorphisms. TGS varied from 0 to 100 (Table 5). At the same time, the most common TGS was 50, in 56 people with a frequency of 0.56. In the study sample, TGS was determined to be equal to 0 with a frequency 0.03 (in 3 people). A low value (TGS = 25) was determined in 17 people, and a high value (TGS = 75) in 22 athletes. The highest result (TGS = 100) was noted only in 2 athletes (frequency 0.04).

In Group I with high qualifications, the TGS calculated for the quality of “endurance” varied from 0 (for 1 athlete) to 100 (for 2 athletes). The most common TGSS was 50 in 31 people. In Group II with low qualifications, the TGS equal to 50 prevailed (for 25 athletes). However, in Group II, the TGS varied from 0 (in 2 athletes) to 75 (in 8 athletes). TGS equal to 100 was not found in Group II with high qualifications.

**Figure 3:**
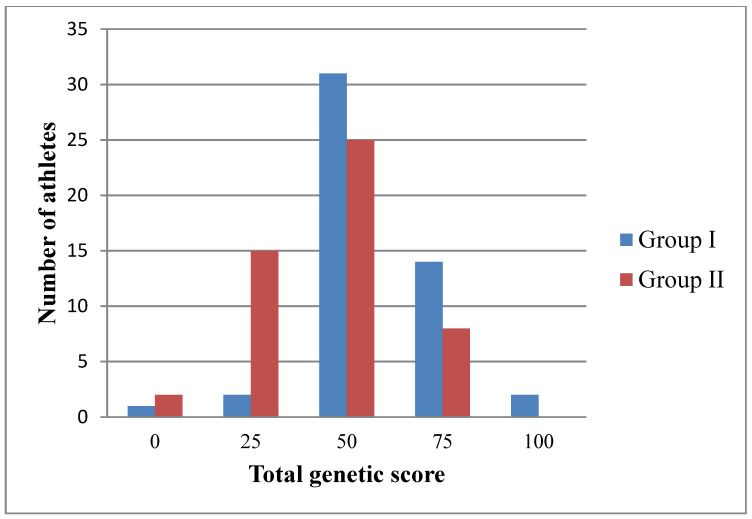
Distribution of the total genetic score established in combatants (*n* = 100) for the quality of “endurance”, based on the polymorphism of the *NOS3* and *PPARGC1A* genes.

Figure 3 shows the distribution of the TGS indicator determined for the development of the “endurance” quality in a sample of 100 martial arts practitioners, based on the polymorphism of the *NOS3* and *PPARGC1A* genes. It is shown that the lowest TGS equal to 0, as well as low TGS equal to 25, are more often noted in Group II with low qualifications. At the same time, the average TGS indicator is 50; high TGS equal to 75; as well as the highest TGS of 100 predominates in Group I with high qualifications.

The Fisher’s test was used to estimate the differences between two samples of martial arts athletes with different sports qualifications—the total genetic score between the groups with high and low qualifications (Table 5). A significant difference was established with a TGS of 25: in group I with a high qualification of 2 individuals and in group II with a low qualification of 15 individuals (F_exp_: 3.63 > 1.96, *p* = 0.05). In addition, the difference in the highest score (TGS = 100) was high but not significant: in Group I it was found in 2 athletes, in Group II there were no athletes with this score (F_exp_: 1.93 < 1.96, *p* = 0.05). In all other cases, there were no significant differences between the TGS values in group I and group II.

Using a chi-square test, statistical significance was determined between the two samples (Group I with high skill and Group II with low skill) and the TGS value. The value of the criterion χ2_emp_ is 14.554, and the critical value of χ2_cr_ at the significance level *p* = 0.01 is 13.277. Significance level *p* = 0.006. Thus, the relationship between the two groups with different qualifications and the value of TGS determined based on the polymorphism of the *NOS3* and *PPARGC1A* genes, is statistically significant at a significance level of *p* < 0.01.

## 4. Discussion

When solving the problems of athletic recruitment, very often incorrect predictions are made about the success of individual athletes. This happens despite the vast experience of teachers and coaches. Modern methods of sports genetics can help to solve this problem, as they help to avoid many unfortunate decisions in this matter with the help of genetic markers [16].

Analysis of the genotype distribution in the high and low skill groups showed that the I/D genotype of the *ACE* gene was more common in group I with a frequency of 0.50. In addition, that I allele is associated with endurance, and the D allele is associated with speed and strength qualities. Thus, we can say that highly skilled athletes from Group I have genotypes that are more balanced in both physical qualities.

Individuals with genotype I/I of the *ACE* gene are characterized by a higher relative content of “slow” muscle fibres and low content of “fast” fibres compared to those with genotype D/D [26]. This fact confirms the role of the I/D polymorphism of the *ACE* gene in the determination of local and general physical performance. A high correlation has been established between an increase in the mass of the left ventricle of the heart after endurance training with an increased level of *ACE* (angiotensin-converting enzyme) in the blood and the D/D genotype. During strength training of the quadriceps femoris muscle, an association of its strength with the D allele of the *ACE* gene was established. In addition, in individuals with the D/D genotype, the ratio of “slow” and “fast” muscle fibres is approximately the same, while in I/I individuals, “slow” muscle fibres dominate [27]. Thus, carriers of the D/D genotype of the *ACE* gene are advised to avoid performing long and heavy physical exertion. In this sample, athletes with the D/D genotype were in the majority—45 people. 43 athletes had the most “balanced” I/D genotype.

In Group II, the genotype +9/+9 of the *BDKRB2* (frequency 0.64) prevailed in relation to the genotype +9/−9 (frequency 0.36). It has been established that carriage of the +9/+9 genotype is accompanied by a higher level of blood pressure, therefore, from this point of view, the +9 allele is considered unfavourable. However, the –9 allele has the opposite meaning and is associated with the lowest HR values.

Allele −9 bp is also associated with high parameters of muscle contraction efficiency; it is associated with arterial hypertension. In this sample of athletes, carriers of the −9/−9 genotype were not identified. It was established [28] that in one study, male swimmers with the +9/+9 genotype had more improvements in swimming than swimmers with the +9/−9 genotype. Thus, it can be assumed that the presence of the +9 allele also positively affects the modulation of the response to long-term training.

Analysis of the Gly/Ser polymorphism of the *PPARGC1A* gene showed that the differences in the Gly/Gly and Gly/Ser genotypes between the groups with high and low qualifications are significant. The 482Ser allele is associated with a decrease in the expression level of the *PPARGC1A* gene, a decrease in oxidative processes and mitochondrial biogenesis, and obesity in men leading an inactive lifestyle [29]. In addition, the Ser allele is associated with an increased risk of developing diabetes mellitus [30], while the Gly allele is associated with high physical performance [31]. Of the 100 subjects in this study, 68 people have the Gly/Gly genotype, and 25 athletes have the Gly/Ser genotype. Only 6 wrestlers have the Ser/Ser genotype. Gly/Gly and Gly/Ser genotypes predominate in both groups, and the most favourable Gly/Gly genotype occurs with a frequency of 0.80 in the group of highly qualified athletes (F_exp_: 2.32 > 1.96, *p* = 0.05). Thus, we can conclude that the Gly allele is significant among combat athletes.

In determining the polymorphism G894T of the *NOS3* gene in 100 athletes from Perm, engaged in martial arts, it was found that the frequency of genotype G/G is significantly higher in group I with high qualification (F_exp_ 3.39 > 1.96, *p* = 0.05). This genotype is characterized by high activity of endothelial *NO*-synthase and a high level of production of nitric monoxide, thus the balance of production of endothelial vasoactive factors is not disturbed. The G/T genotype, which corresponds to the average activity of endothelial *NO*-synthase and the average level of production of nitric oxide, as a result of which the balance of production of endothelial vasoactive factors is disturbed, was detected in 28 people with a slight difference in both groups (in Group I, with a frequency of 0.30 people, in Group II-frequency 0.26). The most unfavourable T/T genotype, in which there is low activity of endothelial *NO*-synthase and a decrease in the production of nitric monoxide, as a result of which the balance of production of endothelial vasoactive factors is disturbed, was found in the majority of subjects, that is, in 66 athletes. Carriers of this genotype are recommended a moderate pace of exercise.

Martial arts are characterized by different power tension and energy consumption, which depend on the dynamism of the fight and the weight of the opponent. In addition, in wrestling, quick reactions to the opponent’s actions and endurance are of great importance. In this sample, athletes with genotypes I/D (frequency 0.43) and D/D (frequency 0.45) of the *ACE* gene, +9/+9 (frequency 0.57) of the *BDKRB2* gene, T/T (frequency 0.66) gene *NOS3*, Gly/Gly (frequency 0.68) gene *PPARGC1A*. Based on these data, we can conclude that in martial arts there are athletes who are predisposed to the development of endurance and speed-strength qualities. Carriers of genotypes I/I of the *ACE* gene (frequency 0.12), Gly/Gly of the *PPARGC1A* gene (frequency 0.68), and G/G of the *NOS3* gene (frequency 0.06) are predisposed to performing long-term physical work. Carriers of these genotypes are allowed endurance exercises, while it is recommended to reduce the speed-strength load.

No significant differences were found in the allele and genotype frequencies of the *ACE* gene I/D polymorphism in patients with arterial hypertension compared to controls in most studies [9]. However, some studies have shown the dependence of the D/D genotype in men with high blood pressure [32,33]. Allele −9 of the *BDKRB2* gene is associated with arterial hypertension [12,15]. Thus, carriers of the D/D genotype of the male *ACE* gene (there were 37 people in this sample) and carriers of the −9 genotype of the *BDKRB2* gene are recommended to prevent cardiovascular diseases, mainly arterial hypertension.

The most significant genotype of the *ACE* gene of the renin-angiotensin system turned out to be a D/D genotype associated with speed and strength qualities. It occurs with a frequency of 0.45 among the athletes in this sample. Compared to other genes, we can see that G/G genotypes of the NOS3 gene and Gly/Gly genotypes of the *PPARGC1A* gene associated with physical quality “endurance” are also significantly more frequent in the group of highly qualified athletes. Many athletes have genotypes associated with speed and strength qualities: T/T of the *NOS3* gene (frequency 0.66) and Gly/Ser of the *PPARGC1A* gene (frequency 0.25).

The results of this study showed that in this sampling of martial athletes from the city of Perm there is a statistically significant relationship between the TGS for the quality of “endurance” in group I with high qualification and group II with low qualification. Analysis of *ACE* and *BDKRB2* gene profiles of the examined athletes engaged in martial arts revealed the athletes most predisposed to the manifestation of the “endurance” quality. It was found that 5 athletes have a high TGS equal to 75, defined as the physical quality “endurance” (frequency 0.05). An average value of TGS equal to 50 was found in 22 athletes (frequency 0.22). A low TGS value of 25 was found in 51 individuals (frequency 0.51). The lowest TGS equal to 0 was detected in 22 athletes (frequency 0.22). The highest TGS of 100 was not found in the study sample. Compared to the TGS determined based on *NOS3* and *PPARGC1A* gene polymorphisms, highly skilled Group I athletes were also more likely to have the highest TGS, in contrast to the low-skilled group of athletes, where TGS equal to 0 and 25 were more frequent.

Thus, the results of this study showed that the analysis of the genetic profile makes it possible to identify promising athletes who respond positively to physical activity, as opposed to athletes for whom such activity is undesirable.

Based on the findings of molecular genetic analysis of polymorphic gene variants associated with the physical quality “endurance”, individual genotyping reports were compiled for each athlete and shared with the athletes and their coaches. The information obtained can help coaches to design individual training programs for athletes to improve the effectiveness of the training process. Athletes with unfavourable alleles should follow training intensity recommendations to stay healthy.

The following genotypes can serve as markers indicating high performance in martial arts selection: the D/D genotype of the *ACE* gene associated with the physical quality “speed/strength”, the Gly/Gly genotype of the *PPARGC1A* gene, and the G/G genotype of the *NOS3* gene associated with the physical quality “endurance”. At the same time, carriers of the T/T genotype (frequency 0.66) of the *NOS3* gene are recommended a moderate exercise rate because they have reduced *NO* synthase activity. For athletes with the Gly/Ser and Ser/Ser genotypes of the *PPARGC1A* gene, alternating load and rest during exercise are recommended, because carriers of this genotype have reduced aerobic performance. For athletes with high TGS (75 and 100 points) on the physical quality “endurance”, intensive training loads are possible. For athletes with an average TGS (50 points) on the physical quality “endurance”, a load of moderate intensity is recommended. Particular attention is paid to athletes with low TGS (0 and 25 points) in “endurance” quality, because the intensity of the load during training should increase gradually and dimensionally, and the duration of the session as a whole should not exceed 30–60 min.

## 5. Conclusions

The data obtained prove that athletes with the highest TGS are the most highly skilled. At the same time, athletes with unfavourable genotypes are excluded from the recruitment process, because they do not develop endurance to the extent necessary for martial arts. This happens for many reasons, one of which is their low performance due to genetics and long recovery times after training. Genetic analysis data will be useful in the process of training martial arts athletes in sports schools, because they can be used to design individual training programs, taking into account the genotypes and physical fitness features of a particular athlete. This should lead to an increase in sports achievements, preservation of health and increased sports longevity [16].

Polymorphic loci of four genes whose products are involved in blood pressure control (*ACE*, *BDKRB2*, *NOS3* and *PPARGC1A*) can be used in martial arts not only to determine predisposition to cardiovascular disease but also to predispose to the development of speed and power qualities and endurance. TGS can serve as a tool for predicting athletic success.

## Figures and Tables

**Table 1:** Primers for amplification of polymorphic loci of four genes in martial arts practitioners.

Gene/Polymorphic Locus *	Primer Sequence (5′-3′)	Tm (°C) **	Primer Quality (%)	References
*ACE/I/D*	CTGGAGACCACTCCCATCCTTTCT	66.4	83	[25]
	GATGTGGCCTACACATTCGTCAGAT	65.1	66	
*BDKRB2/+9/−9*	TCTGGCTTCTGGGCTCCGAG	67.4	61	[22]
	AGCGGCATGGGCACTTCAGT	68.2	79	
*NOS3/*G894T	AAGGCAGGAGACAGTGGATGGA	66.3	62	[18]
	CCCAGTCAATCCCTTTGGTGCTCA	67.8	86	
*PPARGC1A/*Gly482Ser	GAGCCGAGCTGAACAAGCAC	64.8	76	[24]
	GGAGACACATTGAACAATGAATAGGATTG	62.5	61	

* *ACE* gene (Angiotensin-1 Converting Enzyme) I/D polymorphism; *BDKRB2* (Bradykinin Receptor b2) gene polymorphism +9/−9; *NOS3* (Nitric Oxide Synthase 3) gene G894T polymorphism; *PPARGC1A* gene (Peroxisome Proliferator-Activated Receptor Gamma Coactivator 1-Alpha) Gly482Ser polymorphism. ** Melting temperature (*T_m_*) calculated for oligonucleotide concentration of 200 nM in 55 mM KCl with 1 mM Mg^2+^ [23].

**Table 2:** Frequencies of the genotypes of the *ACE*, *BDKRB2*, *NOS3*, *PPARGC1A* genes in martial arts practitioners with different qualifications (*n* = 100).

Gene	Genotype	Genotype Frequencies in Sports Qualification Groups *		Total (100 Persons)	F_exp_^<^_>_ F_st_
		Group I (High Qualification, 50 Persons)	Group II (Low Qualification, 50 Persons)		
*ACE*	**I/I**	**0.16 (8)**	0.08 (4)	0.12 (12)	1.20 < 1.96
	I/D	0.50 (25)	0.36 (18)	0.43 (43)	1.36 < 1.96
	D/D	0.34 (17)	0.56 (28)	0.45 (45)	**2.14 > 1.96**
*BDKRB2*	+9/+9	0.50 (25)	0.64 (32)	0.57 (57)	1.36 < 1.96
	+9/−9	0.50 (25)	0.36 (18)	0.43 (43)	1.36 < 1.96
*NOS3*	G/G	0.12 (6)	0	0.06 (6)	**3.39 > 1.96**
	G/T	0.30 (15)	0.26 (13)	0.28 (28)	0.43 < 1.96
	T/T	0.58 (29)	0.74 (37)	0.66 (66)	1.63 < 1.96
*PPARGC1A*	Gly/Gly	0.80 (40)	0.58 (29)	0.68 (68)	**2.32 > 1.96**
	Gly/Ser	0.14 (7)	0.36 (18)	0.25 (25)	**2.50 > 1.96**
	Ser/Ser	0.06 (3)	0.06 (3)	0.06 (6)	0.71 < 1.96

* Group I—athletes with high sports categories (1 junior, 2 adults, 3 adults, CMS); Group II—athletes with low sports categories (no category, 2 juniors, 3 junior); F_exp_—Fisher’s F-test, F_st_—standard Fisher’s test equals 1.96 (*p* = 0.05); significant differences are highlighted in bold.

**Table 3:** The total genetic score of combatants is based on *ACE*, *BDKRB2*, *NOS3*, and *PPARGC1A* gene polymorphisms (*n* = 100).

TGS “Endurance” *	Group I, Number of Athletes (Frequency) (50 Individuals)	Group II, Number of Athletes (Frequency) (50 Individuals)	Number of Athletes (Frequency) (100 Individuals)	F_exp_	χ^2^. for *p* < 0.01
12	1 (0.02)	7 (0.14)	8 (0.08)	**2.32 > 1.96**	20.962
25	4 (0.08)	17 (0.34)	21 (0.21)	**3.22 > 1.96**	
37	22 (0.44)	14 (0.28)	36 (0.36)	1.61 < 1.96	
50	12 (0.24)	10 (0.20)	22 (0.22)	0.46 < 1.96	
62	9 (0.18)	2 (0.04)	11 (0.11)	**2.27 > 1.96**	
75	2 (0.04)	0	2 (0.02)	1.93 < 1.96	

* Comparison of unrelated samples (TGS of athletes between Group I with high qualification and Group II with low qualification) was carried out by Fisher’s test, Fisher’s standard F-test is equal to 1.96 (*p* = 0.05). Evaluation of the relationship between two variables (qualification and TSG) was carried out using the chi-square test (χ2); the critical value of χ2 at the significance level *p* = 0.01 is 15.086; significant differences are highlighted in bold.

**Table 4:** The total genetic score of martial arts practitioners is based on ACE and BDKRB2 genes polymorphism (*n* = 100).

TGS “Endurance” *	Group I 50 Individuals	Group II 50 Individuals	Total 100 Individuals	F_exp_	χ^2^. for *p* < 0.05
	Number of Athletes (Frequency)	Number of Athletes (Frequency)	Number of Athletes (Frequency)		
**0**	**6 (0.12)**	16 (0.32)	22 (0.22)	**2.37 > 1.96**	8.001
25	26 (0.52)	25 (0.50)	51 (0.51)	0.19 < 1.96	
50	14 (0.28)	8 (0.16)	22 (0.22)	1.40 < 1.96	
75	4 (0.08)	1 (0.02)	5 (0.05)	1.39 < 1.96	

* Comparison of unrelated samples (TGS of athletes between Group I with high qualification and Group II with low qualification) was carried out by Fisher’s test, Fisher’s F-test standard is 1.96 (*p* = 0.05). Evaluation of the relationship between two variables (qualification and TGS) was carried out using the chi-square test (χ2); the critical value of χ2 at the significance level *p* = 0.05 is 7.815, the significance level *p* = 0.046; significant differences are highlighted in bold.

**Table 5:** The total genetic score of martial arts practitioners is based on *NOS3* и *PPARGC1A* genes polymorphism (*n* = 100).

TGS “Endurance” *	Group I 50 Individuals	Group II 50 Individuals	Total 100 Individuals	F_exp_	χ^2^. for *p* < 0.01
	Number of Athletes (Frequency)	Number of Athletes (Frequency)	Number of Athletes (Frequency)		
**0**	**1 (0.02)**	2 (0.04)	3 (0.03)	0.57 < 1.96	14.554
25	2 (0.04)	15 (0.30)	17 (0.17)	**3.63 > 1.96**	
50	31 (0.62)	25 (0.50)	56 (0.56)	1.16 < 1.96	
75	14 (0.28)	8 (0.16)	22 (0.22)	1.40 < 1.96	
100	2 (0.04)	0	2 (0.02)	1.93 < 1.96	

* Comparison of unrelated samples (TGS of athletes between Group I with high qualification and Group II with low qualification) was carried out by Fisher’s test, Fisher’s F-test standard is 1.96 (*p* = 0.05). Evaluation of the relationship between two variables (qualification and TGS) was carried out using the chi-square test (χ2); the critical value of χ2 at the significance level *p* = 0.01 is 13.277, the significance level *p* = 0.006; significant differences are highlighted in bold.

## Data Availability

Data presented in this article are available at request from the corresponding author.

## Supplementary Materials

The following supporting information can be downloaded at: https://www.mdpi.com/article/10.3390/genes13091677/s1, Figure S1. A fragment of the electrophoregram of the products of amplification of polymorphic alleles of the *ACE* gene in martial artists. Figure S2. A fragment of the electrophoregram of the products of amplification of polymorphic alleles of the *BDKRB2* gene in martial artists. Figure S3. A fragment of the electrophoregram of the products of amplification of polymorphic alleles of the *NOS3* gene in martial artists. Figure S4. A fragment of the electrophoregram of the products of amplification of polymorphic alleles of the *PPARGC1A* gene in martial artists. Table S1. The main characteristics of the combatants of Group I and Group II; Table S2. Data on tested athletes
