# Supplementary material for: Gene Polymorphism and Total Genetic Score in Martial Arts Athletes with Different Athletic Qualifications
Source: Genes (Basel). 2022 Sep 19;13(9):1677. doi: 10.3390/genes13091677 (PMC9498734; doi:10.3390/genes13091677)
Supplement: Supplementary file 1 [file genes-13-01677-s001.zip › genes-1922216-supplementary.pdf]

Supplementary Material

Gene polymorphism and total genetic score in martial arts athletes with different athletic qualifications

Anna Vostrikova [1], Victoria Pechenkina [1], Maria Danilova [1], Svetlana Boronnikova [1*], Ruslan Kalendar [2,3*]

[1] Faculty of Biology, Perm State National Research University, 614990, Perm, Russia;

[2] National Laboratory Astana, Nazarbayev University, Nur-Sultan 010000, Kazakhstan;

[3] Institute of Biotechnology HiLIFE, University of Helsinki, 00029 Helsinki, Finland;

* Authors to whom correspondence should be addressed: svboronnikova@yandex.ru (SB); ruslan.kalendar@helsinki.fi (RK); Tel.: +358294158869 (RK)

AV: vostanyuta@mail.ru ORCID 0000-0002-9330-2918

VP: p_viktoria2@mail.ru ORCID 0000-0003-4241-4482

MD: ruadry@list.ru ORCID 0000-0002-1916-9709

SB: svboronnikova@yandex.ru ORCID 0000-0002-5498-8160

RK: ruslan.kalendar@helsinki.fi ORCID 0000-0003-3986-2460

Fragments of the electrophoregram of PPARGC1A, NOS3, BDKRB2, ACE gene amplification products

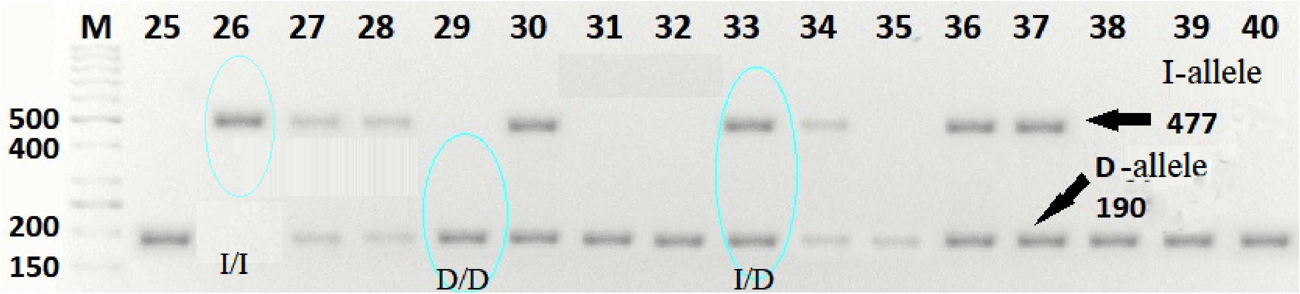

Figure S1. A fragment of the electrophoregram of the products of amplification of polymorphic alleles of the *ACE* gene in martial artists; I/I, I/D, D/D - genotypes of the *ACE* gene; the numbers at the top indicate the serial numbers of the samples; M - molecular weight marker (100 bp + 1.5 + 3 Kb DNA Ladder); arrows indicate alleles; represented part of the spectrum.

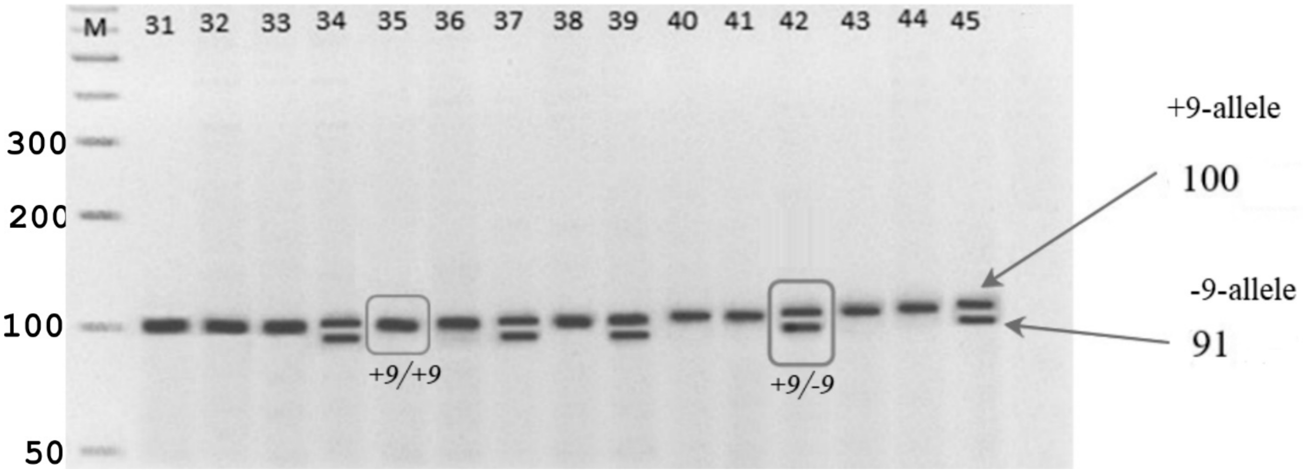

Figure S2. A fragment of the electrophoregram of the products of amplification of polymorphic alleles of the *BDKRB2* gene in martial artists; +9/+9, -9/+9, *BDKRB2* gene genotypes; the numbers at the top indicate the serial numbers of the samples; Size marker: 50 bp, 100 bp DNA Ladder, marked on the left in bp (50 bp to 500 bp); arrows indicate alleles.

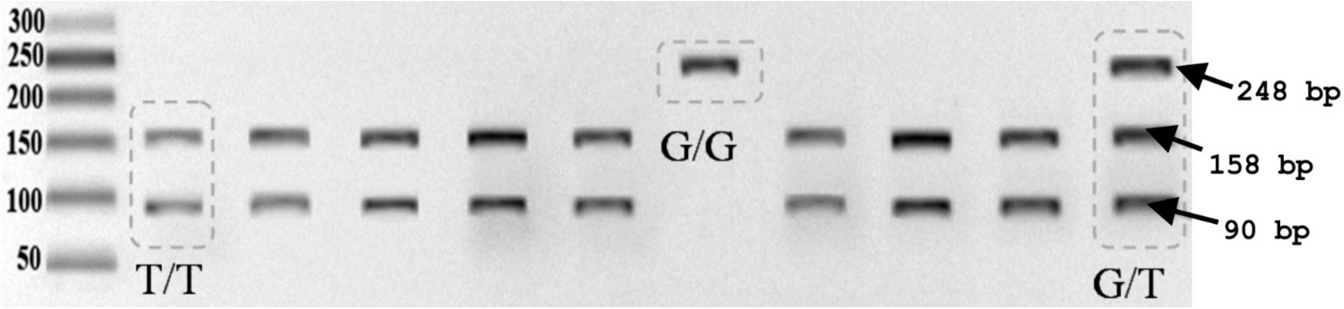

Figure S3. A fragment of the electrophoregram of the products of amplification of polymorphic alleles of the *NOS3* gene in martial artists: G/G, G/T, T/T – genotypes of the *NOS3* genes, numbers indicate the sample numbers, M – molecular marker (50 bp DNA Ladder), alleles are indicated by arrows.

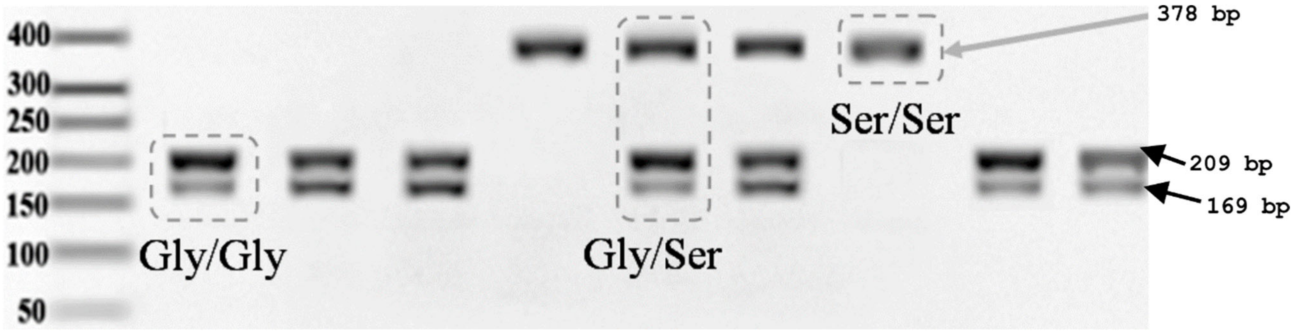

Figure S4. A fragment of the electrophoregram of the products of amplification of polymorphic alleles of the *PPARGC1A* gene in martial artists: Gly/Gly, Gly/Ser, Ser/Ser - *PPARGC1A* gene genotypes, numbers indicate sample numbers, M - molecular marker (50 bp DNA Ladder), alleles are indicated by arrows.

Table S1. The main characteristics of the combatants of Group I and Group II.

Characteristics	Group I	Group II
Age	10 - 16 years old<br>(Average age 13 years)	10 - 16 years old<br>(Average age 12 years)
Male gender	39 athletes	43 athletes
Female gender	11 athletes	7 athletes
Qualification	1 youth categories: 29 athletes<br>3 adult categories: 11 athletes<br>2 adult categories: 4 athletes<br>CMS: 6 athletes	2 youth categories: 23 athletes<br>3 youth categories: 10 athletes<br>no categories: 17 athletes
Total number of athletes	50	50

Table S2. Data on tested athletes.

№	Gender	Age	Qualification	Sports experience	Group	*ACE*	*BDKRB2*	*PPARGC1A*	*NOS3*	TGS "endurance"
1	female	13	1 youth	5	I	I/D	+9/+9	Gly/Gly	T/T	37
2	male	12	1 youth	4	I	I/D	+9/+9	Gly/Gly	T/T	37
3	male	14	3 youth	4	II	I/D	-9/+9	Gly/Gly	T/T	50
4	male	11	3 youth	3	II	I/D	-9/+9	Ser/Ser	T/T	25
5	male	13	1 youth	5	I	I/I	-9/+9	Gly/Gly	T/T	62
6	male	12	1 youth	5	I	I/D	+9/+9	Gly/Gly	G/T	50
7	male	13	2 youth	3	II	I/D	+9/+9	Gly/Gly	T/T	37
8	male	13	1 youth	5	I	D/D	+9/+9	Gly/Gly	T/T	25
9	male	15	-	2	II	I/D	-9/+9	Gly/Gly	G/T	62
10	male	12	1 youth	5	I	I/D	+9/+9	Gly/Gly	T/T	37
11	male	15	3 adults	6	I	I/D	-9/+9	Gly/Gly	T/T	50
12	male	13	1 youth	4	I	I/I	+9/+9	Gly/Gly	G/T	62
13	male	11	1 youth	4	I	I/D	+9/+9	Gly/Gly	T/T	37
14	male	11	1 youth	4	I	I/D	+9/+9	Gly/Gly	T/T	37
15	female	13	1 youth	6	I	I/D	-9/+9	Gly/Gly	G/T	62
16	male	11	2 youth	2	II	I/D	+9/+9	Gly/Gly	T/T	37
17	female	11	1 youth	3	I	I/D	-9/+9	Gly/Gly	T/T	50
18	female	10	2 youth	3	II	I/D	+9/+9	Gly/Gly	G/T	50
19	male	12	1 youth	4	I	I/D	+9/+9	Gly/Gly	G/T	50
20	male	16	1 youth	5	I	I/I	-9/+9	Gly/Gly	G/T	75
21	male	13	1 youth	3	I	I/D	+9/+9	Gly/Gly	T/T	37
22	male	14	1 youth	4	I	I/D	+9/+9	Gly/Gly	T/T	37
23	female	11	1 youth	3	I	I/D	-9/+9	Gly/Gly	G/T	62
24	male	13	2 youth	2	II	I/D	+9/+9	Gly/Gly	T/T	37
25	male	11	2 youth	3	II	I/I	-9/+9	Gly/Ser	T/T	50
26	male	10	2 youth	2	II	I/D	-9/+9	Gly/Gly	T/T	50
27	female	10	1 youth	3	I	I/I	+9/+9	Gly/Gly	T/T	50
28	male	16	CMS	9	I	I/D	+9/+9	Gly/Gly	T/T	37

29	female	14	2 adults	7	I	I/D	+9/+9	Gly/Gly	T/T	37
30	male	14	2 adults	6	I	D/D	+9/+9	Gly/Gly	G/T	37
31	male	14	2 adults	7	I	I/D	+9/+9	Gly/Gly	G/G	62
32	male	16	3 adults	6	I	I/D	-9/+9	Gly/Ser	G/G	62
33	male	15	3 adults	5	I	I/D	+9/+9	Gly/Ser	G/G	50
34	male	16	3 adults	7	I	I/D	-9/+9	Gly/Ser	T/T	37
35	male	16	3 adults	6	I	I/D	+9/+9	Gly/Ser	T/T	25
36	male	14	2 youth	5	II	D/D	-9/+9	Gly/Ser	T/T	25
37	male	14	2 youth	6	II	D/D	+9/+9	Gly/Ser	G/T	25
38	male	13	2 youth	5	II	D/D	+9/+9	Gly/Ser	T/T	12
39	male	10	2 youth	3	II	D/D	+9/+9	Gly/Ser	T/T	12
40	female	12	2 youth	5	II	D/D	+9/+9	Gly/Ser	T/T	12
41	female	12	2 youth	4	II	D/D	+9/+9	Gly/Ser	T/T	12
42	female	13	1 youth	5	I	D/D	-9/+9	Gly/Ser	G/T	37
43	male	12	1 youth	4	I	D/D	-9/+9	Gly/Gly	T/T	37
44	male	13	1 youth	4	I	D/D	+9/+9	Gly/Gly	G/T	37
45	male	12	1 youth	4	I	D/D	+9/+9	Gly/Gly	T/T	25
46	male	13	1 youth	5	I	D/D	-9/+9	Gly/Gly	G/T	50
47	male	12	1 youth	4	I	D/D	-9/+9	Gly/Ser	G/T	37
48	male	13	1 youth	5	I	D/D	-9/+9	Gly/Gly	T/T	37
49	male	11	1 youth	3	I	D/D	+9/+9	Gly/Gly	G/T	37
50	male	11	1 youth	4	I	D/D	-9/+9	Gly/Gly	T/T	37
51	male	15	-	2	II	D/D	-9/+9	Gly/Ser	T/T	25
52	male	12	-	1	II	D/D	-9/+9	Gly/Ser	T/T	25
53	male	12	-	2	II	D/D	-9/+9	Gly/Ser	T/T	25
54	male	11	3 youth	1	II	D/D	-9/+9	Gly/Ser	G/T	37
55	male	11	1 youth	3	I	D/D	-9/+9	Gly/Gly	T/T	37
56	male	13	-	1	II	D/D	+9/+9	Gly/Ser	T/T	12
57	male	14	-	2	II	D/D	+9/+9	Gly/Ser	G/T	25
58	male	13	-	2	II	D/D	-9/+9	Gly/Ser	T/T	25
59	male	13	-	1	II	I/D	+9/+9	Gly/Ser	T/T	25
60	female	13	1 youth	5	I	D/D	-9/+9	Gly/Ser	G/T	37

61	male	12	-	2	II	D/D	+9/+9	Gly/Gly	G/T	37
62	male	12	-	2	II	I/D	+9/+9	Gly/Ser	T/T	25
63	male	15	-	2	II	D/D	-9/+9	Gly/Gly	G/T	50
64	male	12	-	1	II	D/D	+9/+9	Gly/Gly	T/T	25
65	male	14	-	2	II	I/D	+9/+9	Gly/Gly	T/T	37
66	female	14	2 youth	5	II	I/D	+9/+9	Gly/Gly	T/T	37
67	male	15	-	2	II	D/D	+9/+9	Gly/Gly	G/T	37
68	male	16	-	2	II	I/D	+9/+9	Gly/Gly	T/T	37
69	male	11	3 youth	3	II	I/D	+9/+9	Gly/Gly	G/T	50
70	male	13	-	2	II	D/D	+9/+9	Gly/Gly	T/T	25
71	female	11	1 youth	4	I	D/D	-9/+9	Gly/Gly	G/T	50
72	male	10	3 youth	2	II	D/D	+9/+9	Gly/Ser	T/T	12
73	male	10	-	1	II	D/D	+9/+9	Gly/Gly	T/T	25
74	female	11	3 youth	3	II	D/D	-9/+9	Gly/Gly	T/T	37
75	male	10	2 youth	2	II	D/D	+9/+9	Gly/Gly	T/T	25
76	male	10	3 youth	3	II	D/D	+9/+9	Gly/Gly	T/T	25
77	male	16	CMS	9	I	I/D	-9/+9	Gly/Gly	G/T	62
78	male	10	2 youth	2	II	I/I	+9/+9	Gly/Gly	G/T	62
79	male	10	2 youth	3	II	I/D	+9/+9	Gly/Gly	T/T	37
80	male	10	2 youth	2	II	I/I	+9/+9	Gly/Gly	T/T	50
81	male	16	3 adults	6	I	I/I	+9/+9	Gly/Gly	T/T	50
82	male	10	2 youth	2	II	I/I	+9/+9	Gly/Gly	T/T	50
83	female	16	CMS	8	I	I/D	+9/+9	Ser/Ser	T/T	12
84	female	16	CMS	9	I	I/D	-9/+9	Gly/Gly	G/G	75
85	male	16	CMS	9	I	I/D	-9/+9	Gly/Gly	T/T	50
86	male	16	CMS	9	I	D/D	-9/+9	Ser/Ser	G/G	37
87	male	12	2 youth	3	II	I/D	+9/+9	Gly/Gly	T/T	37
88	male	13	2 youth	4	II	D/D	-9/+9	Gly/Gly	G/T	50
89	male	16	3 adults	6	I	D/D	-9/+9	Ser/Ser	G/G	37
90	male	15	3 adults	6	I	I/I	-9/+9	Gly/Gly	T/T	62
91	male	14	2 adults	6	I	I/D	-9/+9	Gly/Gly	T/T	50
92	male	15	3 adults	5	I	I/I	+9/+9	Gly/Gly	T/T	50

93	male	16	3 adults	7	I	D/D	+9/+9	Gly/Gly	T/T	25
94	female	12	2 youth	3	II	I/D	-9/+9	Gly/Gly	T/T	50
95	male	15	3 adults	7	I	I/I	-9/+9	Gly/Gly	T/T	62
96	male	10	3 youth	2	II	D/D	-9/+9	Gly/Ser	G/T	37
97	male	11	2 youth	3	II	D/D	-9/+9	Gly/Gly	T/T	37
98	male	11	3 youth	2	II	I/D	+9/+9	Ser/Ser	G/T	25
99	female	13	3 youth	4	II	D/D	-9/+9	Ser/Ser	T/T	12
100	male	16	2 youth	6	II	D/D	+9/+9	Gly/Gly	T/T	25
